# Transanal Endoscopic-Assisted Pull-Through Colectomy for Children with High Intestinal Aganglionosis

**DOI:** 10.3390/children9050588

**Published:** 2022-04-21

**Authors:** Ulrike Metzger, Armin-Johannes Michel, Mircia-Aurel Ardelean, Roman Patrick Metzger

**Affiliations:** Department of Pediatric and Adolescent Surgery, Paracelsus Medical University Hospital, 5020 Salzburg, Austria; a.michel@salk.at (A.-J.M.); m.ardelean@salk.at (M.-A.A.); r.metzger@salk.at (R.P.M.)

**Keywords:** intestinal aganglionosis, endoscopic assisted, endorectal pull-through, Hirschsprung’s disease

## Abstract

Intestinal aganglionosis in children is a common cause of neonatal and infantile obstruction or ileus. Diagnosis is based on a histologically proven absence of enteric ganglion cells in deep biopsies of the gut wall. Therapeutic goal is a one-stage repair with a resection of the affected segment. The endorectal pull-through (ERP) can be performed entirely transanally in a lot of the cases. In patients with difficult preparation or a high aganglionosis ERP often needs to be assisted by laparoscopy or laparotomy. We present two cases with a technical modification performing a totally transanal pull-through colectomy without any trocars other than an umbilical camera trocar. The procedure starts with a classical endorectal technique. Usually, the transanal preparation is limited by reaching the colon descendens. A camera trocar is inserted and under laparoscopic vision the preparation is completed placing the instruments directly via the opened anus. After reaching the healthy colon segment, the pull-through is completed transanally. One of the main advantages of ERP is the sparing dissection. Our modification combines advantages of laparoscopy and ERP. The umbilical camera allows an excellent view while the instruments for dissection are used like with ERP without any further trocar or traction of the anal sphincter. The dispensation of any transanal trocar allows a higher grade of freedom in preparation and possibly a smaller trauma on the distal anal channel.

## 1. Introduction

Intestinal aganglionosis, so called Hirschspung’s disease (HSCR), in children is a sporadic disease. About 1:5000 newborns in Europe shows this neural crest disease. Classically HSCR is diagnosed in the neonatal period. Symptoms include abdominal distension, vomiting and failure to pass meconium during the first day of life. HSCR needs to be excluded in neonatal and infantile obstruction of the gut and should be considered as a differential diagnosis in children with chronic constipation. About 80 percent of the children with diagnosed HSCR present with a so called “short aganglionosis”, that involves solely the rectosigmoidal gut. 

Associated malformations are quite common, 20 percent of the children show additional congenital anomalies. Genetic analysis may be necessary, particularly in patients with additional congenital abnormalities [1]. Diagnosis is based on rectal biopsies. There are limitations to visualize ganglion cells in neonates and premature infants. This should be considered, and biopsies might be repeated [1,2,3,4,5,6,7]. Additionally, most centers perform a contrast enema to identify the level of the transition zone, also accuracy of this technique is discussed on [8]. Anal manometry may also add information [9]. 

Preoperative management includes the applications of saline rectal irrigations to decompress the affected bowel. The early involvement of the parents in the application of the rectal irrigations as well as a nutritional counseling enables the parents and eases the long-term management of HSCR patients [1,4,5]. Since the beginning of the operative management in the early 1940’s, medical handling of this complex and rare disease gained a lot of improvement in the diagnosis and the surgical management. In nowadays a colostomy is rarely needed because of early diagnosis and consequent preoperative bowel management [6,10]. 

The surgical goal is a one-stage repair with resection of the affected segment, the transanal pull-through and an anastomosis in the anal canal. The most common techniques are transanal endorectal pull-through (ERP) with or without laparoscopic assistance and the Duhamel procedure. Both techniques show equal results in long-term continence outcomes and in the number of the most common complications like impairment of bowel function and enterocolitis [2,6,11,12,13,14]. Minimally invasive surgery has been used for intestinal aganglionosis since more than 20 years [14,15,16]. 

Since then, technical modifications of the classical laparoscopic approach like single trocar laparoscopic assisted pull-through (SILEP) ([17,18,19,20,21,22] or other laparoendoscopic single-site surgery (LESS) [22,23,24,25] and Natural Orifice Translumenal Endoscopic Surgery (NOTES) [25,26] have been published. In SILEP by now mostly a special designed port like the TriPort+™ is used, which allows to use a camera and at least two instruments via one trocar. For NOTES™ the use of cannulas through the muscular cuff [26] or an access through the sigmoid vascular pedicle can be found [27]. Combining NOTES™ and LESS Vadhad et al. describe the use of a TriPort+™ transanally [23,24,25].

The transanal pull-through as published by de La Torre is our preferred technique. In cases of difficult preparation or high-level transition zone we introduced a modified technique based on NOTES™. 

## 2. Method

Usually in our department the classical de La Torre technique is performed. 

**Figure 1:**
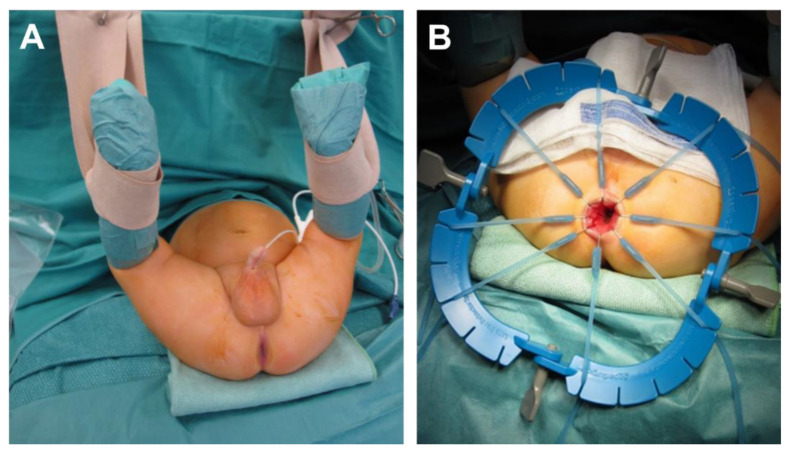
Setting of operation (**A**) and placement of the retractor (**B**), starting classical transanal pull-through.

The patient is positioned in supine position with a slightly elevated pelvis. The anal canal is exposed using a lone star retractor. At 0.5 to 1.0 cm above the dentate line the rectal mucosa is incised circumferentially (Figure 1).

Dissection of the rectal mucosa is performed with a long muscular cuff, up to the level of peritoneal reflection. Now the muscular cuff is divided entering the peritoneal cavity. When the free intraperitoneal plane is achieved, the muscular cuff is divided circumferentially, converting the submucosal dissection into a full-thickness dissection [28]. 

**Figure 2:**
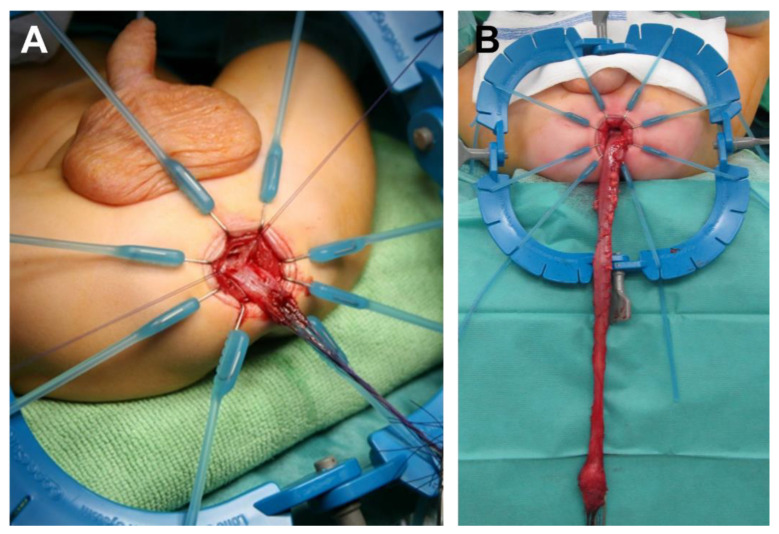
Submucosal preparation (**A**) and full-thickness dissection without entering the transition zone (**B**). This is continued until the transition zone is reached. Full-thickness frozen biopsies are taken immediately cranial to this segment to confirm ganglion cells.

Incision of the posterior wall of the muscular cuff down to the cranial border of the anal sphincter muscle is carried out. The rectum is pulled down and the perirectal and perisigmoid dissection is advanced cranially (Figure 2).

Liberation of the normal colon is proceeded until it reaches the proposed anastomotic line without tension, followed by colectomy of the aganglionic segment. At the end anastomosis is accomplished in the anal canal [28].

**Figure 3:**
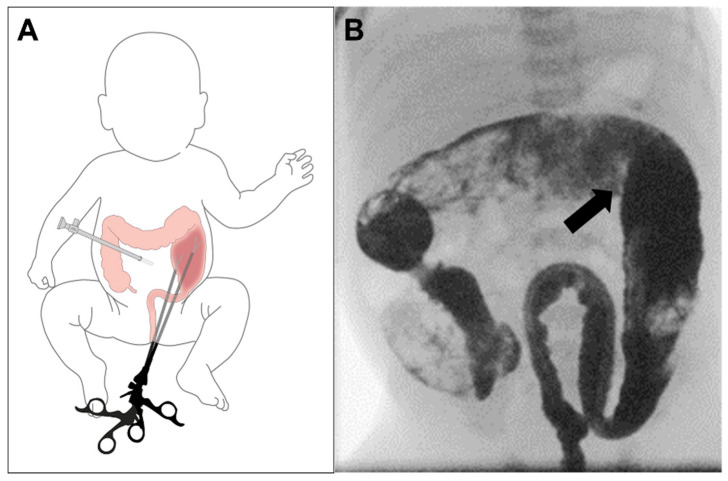
An umbilical camera port is placed, and full-thickness dissection is continued by transanal instrumentation entering the transition zone (**A**). The preoperative abdominal X-ray shows the transition zone (arrow) at the left colonic flexure (**B**).

In some cases of difficult preparation or a long aganglionic segment we expand the standard technique using our modification as follows: 

An umbilical camera trocar is inserted. Pneumoperitoneum is applied with 8 mmHg and a flow of 2 liters. This permits an excellent overview of the abdominal cavity. Then the laparoscopic instruments are inserted in the space between the resected colon and the muscular sleeve without any additional manipulation on the tissue (Figure 3).

**Figure 4:**
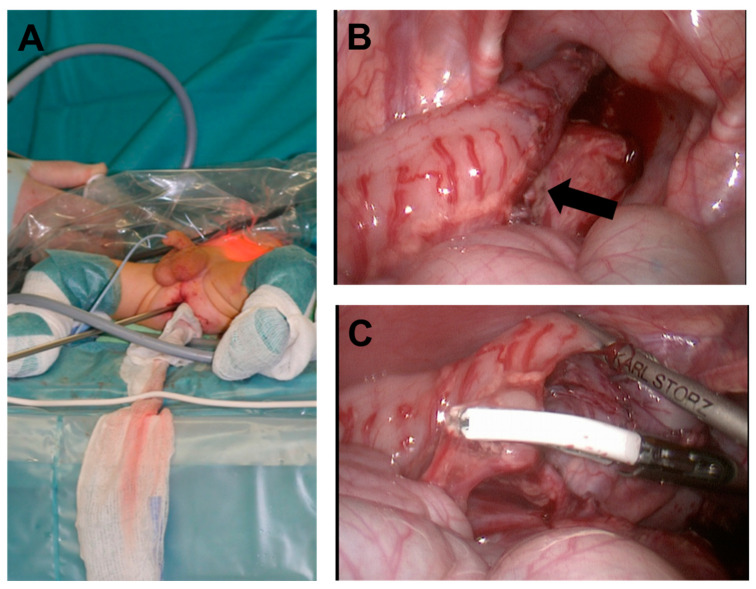
Umbilical camera port is placed, giving an excellent abdominal view (**A**). The transition zone (arrow) is visible now (**B**). Full-thickness dissection is continued by transanal instrumentation entering the transition zone (**C**).

This allows to continue the mobilization in the right layer as described by de La Torre. We use 3- and 5-mm laparoscopic instruments. Instruments like a grasper, but also a 5 mm stapler (JustRight 5 mm stapler, Justright Surgical, LLC, Boulder, CO, USA) or the MiSeal™ (MiSeal™, model 452-131D; Microline, Beverly, MA, USA) are used. The MiSeal^TM^ ensures dissection with minimal thermic harm to the surrounding tissue [29]. If necessary percutaneous application of a 3 mm grasper is possible. (Figure 4).

**Figure 5:**
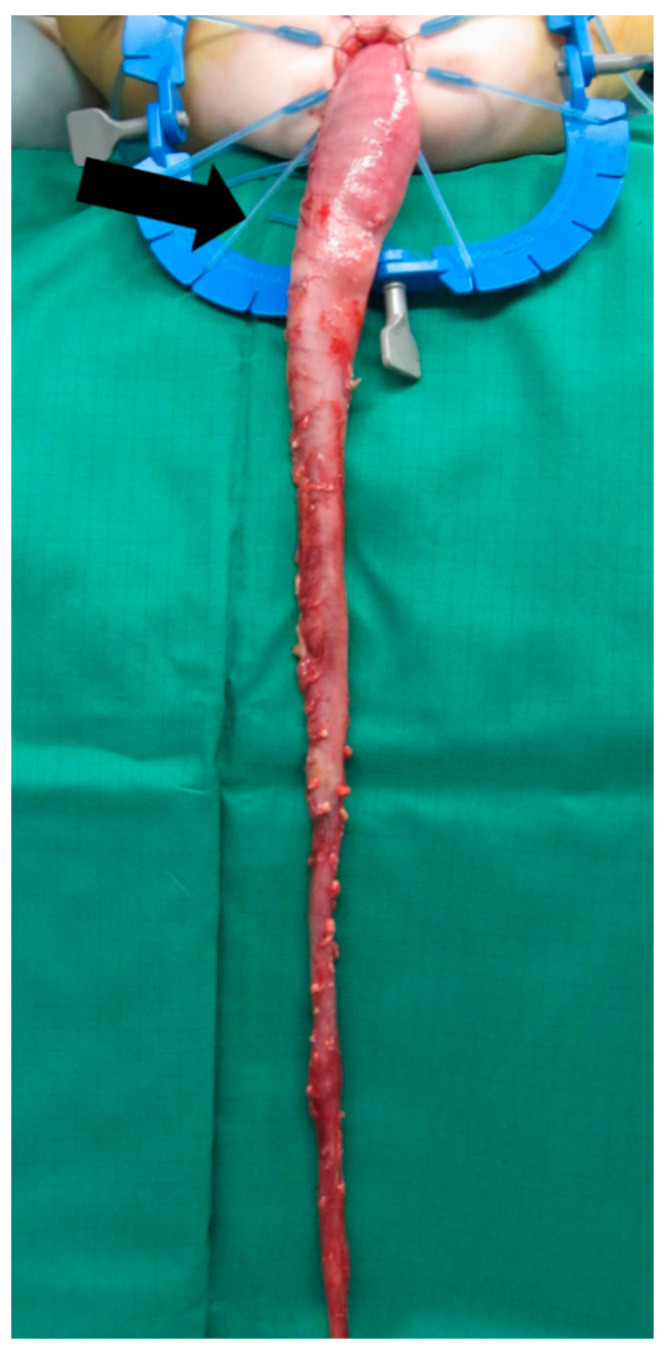
Mobilization of the colon is completed until the transition zone is developed transanally (arrow).

Mobilization of the colon is continued as far as needed to reach the healthy segment. This segment is than freed using the excellent camera view to preserve the important vessels (Figure 5).

Performing the anal anastomosis, the camera allows the control of the correct positioning of the colon.

The postoperative management and follow up are performed according to the guidelines of ERNICA (European Reference Network for rare Inherited and Congenital (digestive and gastrointestinal) Anomalies) [1].

## 3. Results

### 3.1. Case 1

The male newborn was transferred to us at age of 6 days with symptoms of an ileus. Clinical findings and plane radiography confirmed the suspected diagnosis of intestinal aganglionosis. Implementation of bowel management led to fast improvement. Intestinal aganglionosis was proven by repeated biopsies due to initial inconclusive histological findings. Contrast enema showed the suspected transition zone. The newborn was discharged after teaching bowel management to the parents. Informed consent was achieved including laparoscopic assisted pull-through or open surgery.

The transanal endoscopic assisted pull-through colectomy as described above was performed at the age of 5 month. Intraoperatively mobilization was done as far as visually identified transition zone, fresh frozen section biopsies were taken and revealed normal ganglionic colon at about sixty centimeters above the anus. Afterwards ano-descendostomy was conducted. Postoperative course was uncomplicated, and the child could be discharged after 12 days with regular stooling. Calibration started 3 weeks postoperatively and was ended with Charrière (Ch) 12 at about one year of age.

Follow up was performed regularly; this included the involvement of our gastrointestinal pediatricians, ultrasound, and nutritional counseling. By now the boy is 7.5 years old. His bowel function was always regular, and no complications occurred. Cosmetic results are excellent. 

### 3.2. Case 2

Another male newborn was transferred to us at age of 3 days with symptoms of an ileus, vomiting and insufficient meconium defecations. Clinical course was like case 1, after beginning of the bowel management fast improvement was reached and the newborn could be fed with breast milk. Intestinal aganglionosis was proven by biopsies. Also, in this patient contrast enema showed the suspected transition zone. The newborn was discharged after teaching of the bowel management to the parents. 

The transanal endoscopic assisted pull-through colectomy was planned at the age of 3 month. Informed consent was achieved including laparoscopic assisted pull-through or open surgery. Normal ganglionic colon was reached at about forty-five centimeters above the anus. Postoperative course was uncomplicated, and the child could be discharged after 9 days with regular stooling. Calibration was started 3 weeks postoperatively and was realized until about one year of age with Ch 12 in the maximum. 

**Figure 6:**
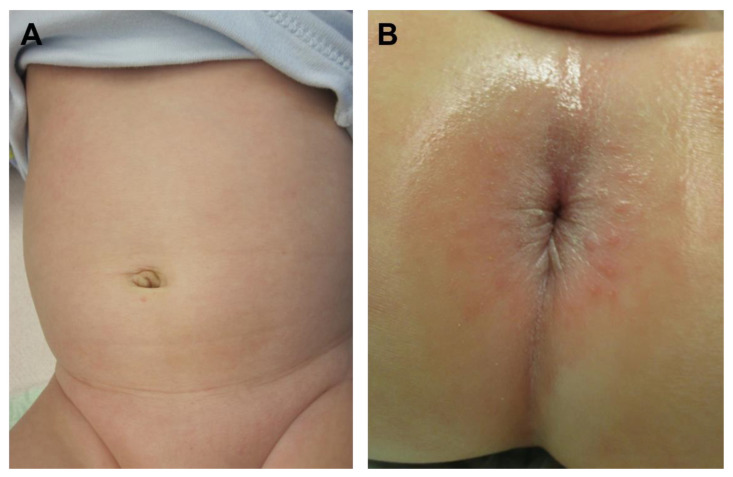
The cosmetic and functional outcome is excellent (**A**). The anal inspection is inconspicuous (**B**).

Follow up of the now 4.5 years old boy was performed regularly, this included the involvement of our gastrointestinal pediatricians, ultrasound, and nutritional counseling. Bowel function was always regular, but the patient complained of flatulence and repeated soft stool. Intolerance of fructose was diagnosed. The patient is now free of symptoms under the implemented diet. Cosmetic results are excellent (Figure 6).

## 4. Discussion

The positive effects as well as the limits of minimally invasive surgery in neonates and infants have been addressed in several papers [30,31,32,33,34,35,36]. One of the goals is to reduce the number of incisions for better cosmetsis. But also, the length of hospital stays, the perioperative morbidity and the costs are affected positively, if the limitations of the minimal access are respected [30,31,32,33,34,35,36]. In repair of HSCR laparoscopic assisted pull-through is introduced since more than 20 years. The completely transanal performance of the pull-through as a modification of the SOAVE technique was first published in the late 1990s by de La Torre [28,37]. All minimal invasive techniques have been pushed forward and a lot of additionally ideas like SILEP or NOTES^TM^ [22,23,24,25,26] have been published in short series or experimental studies. This extensive menu of surgical treatments all show nearly the same results in long term outcome as well as in hospital stay and complications [2,21,32,38].

The SILEP technique allows a laparoscopic assisted mobilization, it permits the surgeon to take biopsies at all needed levels. The affected bowel can be mobilized in nearly the same manner like in classical laparoscopic assisted ERP. It was described initially as an application of more than one trocar or additional instruments via the umbilicus, nowadays primary especially designed ports like TriPort+ ^TM^ are used. Using an additional trocar is described as H-SILEP [22]. Main limitation is the angle of the instruments which is limited and results in a restriction of freedom of the instruments. The TriPort+^TM^ is already quite big in diameter and usually needs incision beyond the navel, but also the application of more than one trocar in the umbilicus may result in traction stress to the tissue. Industrial modifications of the ports trying to solve the limit of the angles has resulted in even bigger ports, which are mostly not feasible for small patients. H-SILEP solves the problem of the angles while having an additional scar and the possibly disadvantages of another abdominal access. Reported results don’t report any difference in the quality or complications compared to classical laparoscopic assisted approach with a benefit for [17,18,19,20,21,22]. 

The modifications using the anal channel with a port as described by Vahdad et al. is reported as a safe method. Access site complications are not reported in the short case series as well as in the performed animal study. The results reported in the case series are only followed up until six months postoperatively. Since functional problems may occur later in follow up there remains the concern, that the maximum stretch to the anal channel may results in an injury of the sphincter muscle [23,24,25]. 

Using cannulas applied through the muscular sleeve results in a direct injury of the distal sleeve. Since scarring is one of the complications of tissue injury, any additional impact on the distal sleeve should be avoided to prevent complications like constipation due to anastomotic narrowness [26]. Another reported case used NOTES^TM^ with an access via the sigmoid vascular pedicle to perform the dissection. This injury of the sigma may result in bacterial translocation to the abdominal cavity and loss of pressure [27].

Main advantage of our modification is the same angle of the instruments likely to de La Torre procedure which allows a close dissection at the aganglionic colon and the release of the ganglionic colon with special focus on the feeding vessels. Additionally, the umbilical camera view adds an excellent abdominal overview and allows dissection with a different angle to the instruments. This enables a safe and high dissection of the colon and ensures a correct positioning of the colon while performing the anastomosis. At least by using the same access as in classical de La Torre procedure, we have no additional manipulation in the anastomotic region. Loss of pneumoperitoneum hasn’t been a problem, since the intact already mobilized colon seals the pneumoperitoneum in the anal channel. 

The modification is limited due to the length and angle of the laparoscopic instruments. This limits the scope from below and may lead to the necessity of additional instrumentation like in laparoscopic assisted transanal pull-through. However, conversion to conventional laparoscopic assisted transanal pull-through is easy to perform. 

We regularly perform contrast enema in our patients preoperatively. The limited accuracy as published by Haikal, et al. [8] hasn’t been a problem so fare since our technique is easy to apply and easy to be converted. The healthy colon is always proven by Fresh frozen biopsies. 

The Management of Hirschsprung’s disease is a complex task which involves not only the pediatric surgeon but the whole center of pediatric health care including experts in histopathology and radiology. Repair is usually possible in a one stage operation. There is more than one comparable option for the surgical repair. As reported before, the main task is maximizing the quality of the performance of the surgical repair and minimizing its complications [38].

## 5. Conclusions

All published technical modifications didn’t led to a change of the gold standard of operating high intestinal aganglionosis using laparoscopic assisted pull-through techniques. 

Our modification emerged from the need of a higher, but still safe dissection while being convinced that the technique of de La Torre is preferrable. One of the main advantages of his technique is the dissection as near as possible to the affected bowel. The classical laparoscopic approaches as well as the techniques using an additional abdominal approach for dissection lack this possibility. On the other side, abdominal overview is best using an umbilical camera trocar. The combination of laparoscopic instruments and the different camera angle results in a technique which allows a lot of freedom for the instruments while keeping a perfect overview of the operated area. The technique is easy to schedule since regular laparoscopic instruments and a plain camera trocar are used. But this technique depends on a high expertise not only in the classically transanal pull-through technique but also in laparoscopic techniques. Also, there might be the need to select patients due to the possible feasibility. This wasn’t needed in our cases since all patients undergo ERP as first line treatment. If dissection cannot be performed easily and free of tension, our technical modification is applied. This could be performed in two patients without any intra- or postoperative complications or the need to convert to standard laparoscopic technique. The presented two boys have excellent cosmetic results and a normal bowel movement with an unsuspicious anastomotic region in ultrasound after more than 4 respectively more than 7 years. 

The modified technique therefore seems to be a feasible additional tool for transanal pull-through of de La Torre allowing highest quality in dissection at least until the transverse colon. Anyhow further research needs to be done in this to prove safety and feasibility.
